# In sync or out of sync?

**DOI:** 10.1007/s12471-024-01918-z

**Published:** 2024-12-23

**Authors:** Benoît Delforge, Lukas Spruyt, Becker S. N. Alzand

**Affiliations:** 1 Department of Internal Medicine, UZ Leuven, Leuven, Belgium; 2 Department of Emergency Medicine, UZ Leuven, Leuven, Belgium; 3 Department of Cardiology, AZ Glorieux, Ronse, Belgium

A 69-year-old woman with a history of hypercholesterolemia and non-obstructive coronary artery disease presented for her annual cardiology follow-up visit. She reported feeling well but mentioned experiencing mild shortness of breath when specifically asked. Clinical examination and echocardiography revealed normal findings. Resting electrocardiogram was performed (Fig. 1).

**Fig. 1:**
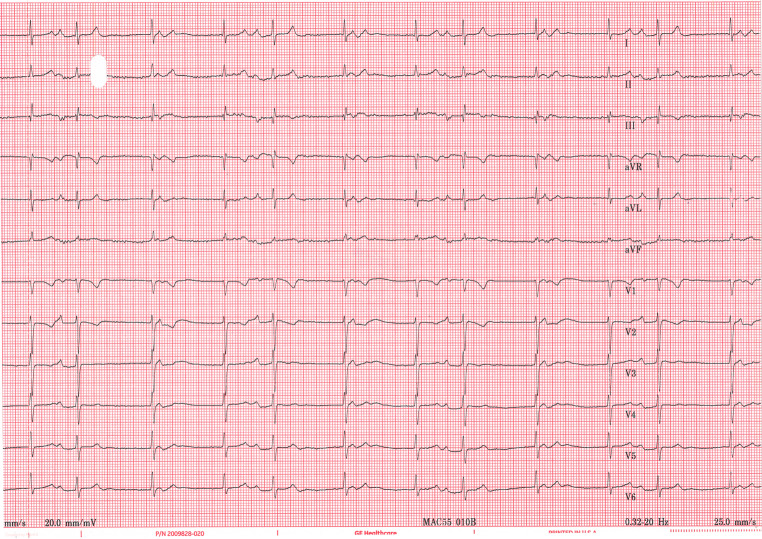
12-lead resting electrocardiogram performed at the outpatient clinic

What rhythm is observed on the electrocardiogram?

## Answer

You will find the answer elsewhere in this issue.

