# Correction to: In sync or out of sync?

**DOI:** 10.1007/s12471-025-01929-4

**Published:** 2025-01-14

**Authors:** Benoît Delforge, Lukas Spruyt, Becker S. N. Alzand

**Affiliations:** 1 Department of Internal Medicine, UZ Leuven, Leuven, Belgium; 2 Department of Emergency Medicine, UZ Leuven, Leuven, Belgium; 3 Department of Cardiology, AZ Glorieux, Ronse, Belgium

**Correction to:**

**Neth Heart J 2024**

10.1007/s12471-024-01918-z

The license information was missing from this article in the online pdf and should have been ‘Open Access This article is licensed under a Creative Commons Attribution 4.0 International License, which permits use, sharing, adaptation, distribution and reproduction in any medium or format, as long as you give appropriate credit to the original author(s) and the source, provide a link to the Creative Commons licence, and indicate if changes were made. The images or other third party material in this article are included in the article’s Creative Commons licence, unless indicated otherwise in a credit line to the material. If material is not included in the article’s Creative Commons licence and your intended use is not permitted by statutory regulation or exceeds the permitted use, you will need to obtain permission directly from the copyright holder. To view a copy of this licence, visit http://creativecommons.org/licenses/by/4.0/.’ The original article has been corrected.

